# Supplementary material for: Hypoxia-inducible factor-1α is a critical transcription factor for IL-10-producing B cells in autoimmune disease
Source: Nat Commun. 2018 Jan 17;9:251. doi: 10.1038/s41467-017-02683-x (PMC5772476; doi:10.1038/s41467-017-02683-x)
Supplement: Supplementary file 1 — Supplementary information [file 41467_2017_2683_MOESM1_ESM.pdf]

Supplementary Figure 1

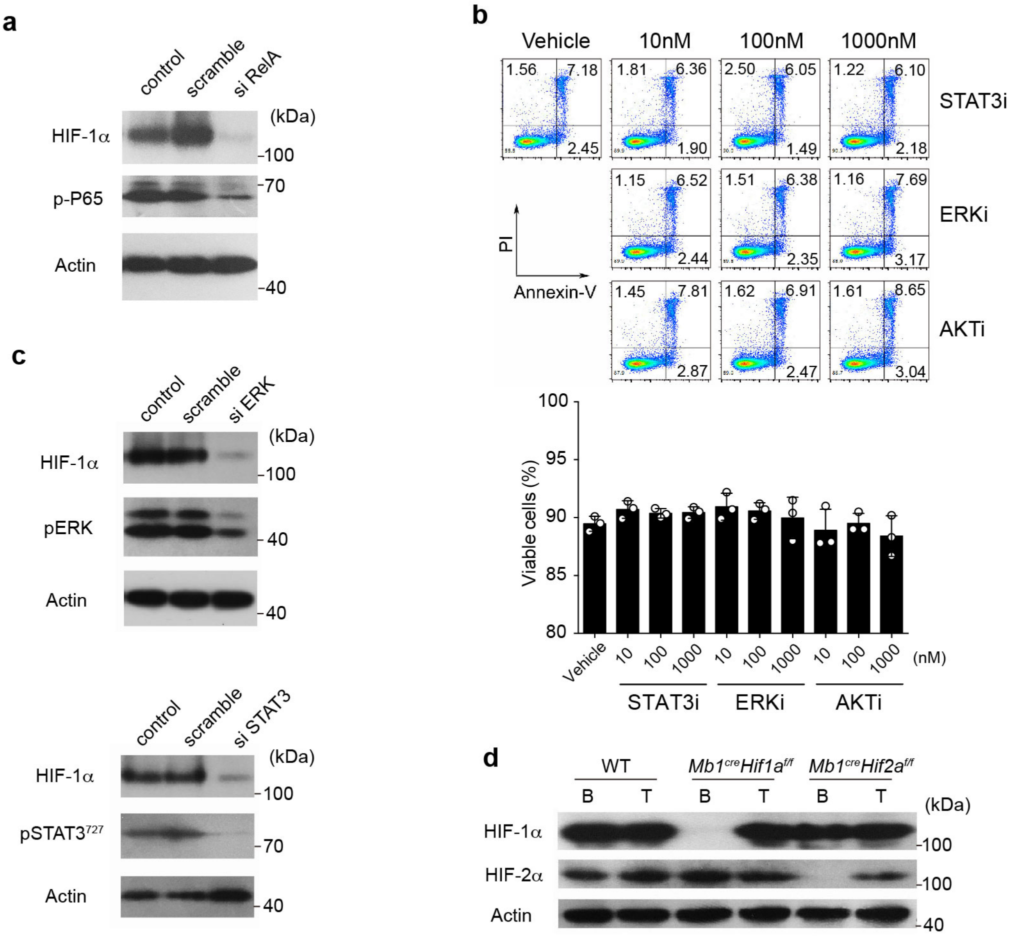

Supplementary Figure 1. Molecular signaling required for HIF-1α expression in activated B cells. (**a**) Western blot analysis of HIF-1α and p-P65 in LPS-stimulated B cells with lentivirus-based RelA siRNA knockdown. (**b**) Annevin-V and PI staining of apoptosis in anti-IgM-stimulated B cells with or without STAT3, ERK or AKT inhibitors treatments for 4 h (n=3 per group). Data represent mean±s.e.m. (**c**) Western blot analysis of HIF-1α, $pSTAT3^{727}$ and pERK in anti-IgM-stimulated B cells with lentivirus-based STAT3 or ERK siRNA knockdown. (**d**) Western blot analysis of HIF-1α, HIF-2α and β-actin in whole-cell lysates of splenic B cells and T cells from WT, $Mb1^{cre}$*Hif1a*$^{f/f}$ and $Mb1^{cre}$*Hif2a*$^{f/f}$ mice, cultured under hypoxic condition for 8 h. Pictures are representative of three independent experiments.

Supplementary Figure 2

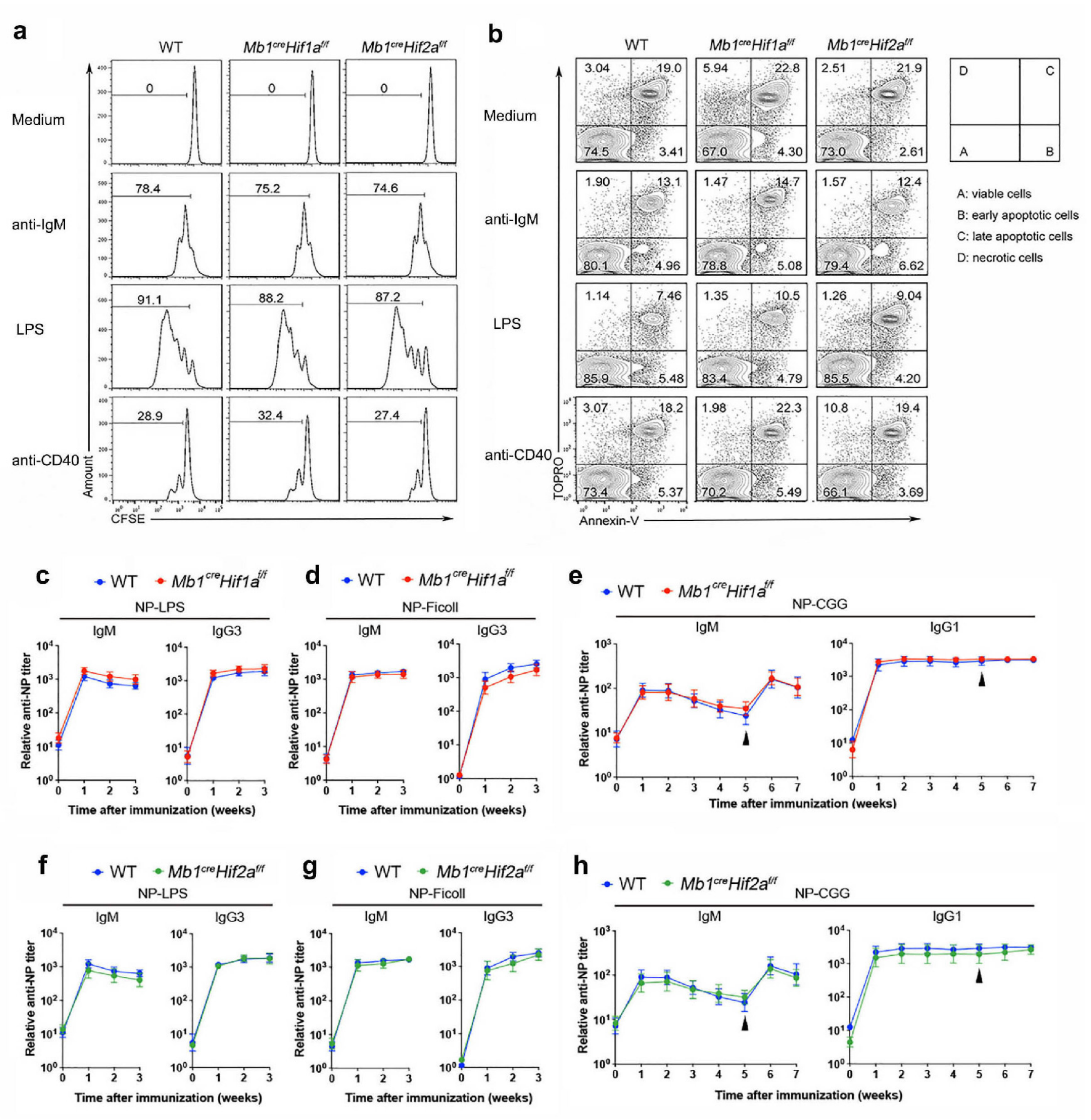

Supplementary Figure 2. Lack of HIF-1α or HIF-2α in B cells has no effect on proliferation, survival and antibody responses. (**a**) Proliferation of enriched splenic B cells from WT, *Mb1^{cre}Hif1a$^{f/f}$* and *Mb1^{cre}Hif2a$^{f/f}$* mice labeled with CFSE and stimulated for 72 h with anti-IgM (10 μg/ml), anti-CD40 (5 μg/ml) or LPS (10 μg/ml). (**b**) Survival of splenic B cells from WT, *Mb1^{cre}Hif1a$^{f/f}$* and *Mb1^{cre}Hif2a$^{f/f}$* mice cultured as described in (**a**), stained with TOPRO and Annexin-V. (**c**,**d**) IgM and IgG3 NP-specific antibody responses of WT littermates and *Mb1^{cre}Hif1a$^{f/f}$* mice after immunization with NP-LPS(**c**) or NP-Ficoll (**d**), as assessed by NP-specific ELISA. (**e**) IgM and IgG1 NP-specific antibody responses of WT littermates and *Mb1^{cre}Hif1a$^{f/f}$* mice after immunization with NP-CGG, boosted with NP-CGG 5 weeks after primary immunization, as assessed by NP-specific ELISA. Arrowheads indicate the day of secondary immunization. (**f**,**g**) IgM and IgG3 NP-specific antibody responses of WT littermates and *Mb1^{cre}Hif2a$^{f/f}$* mice after immunization with NP-LPS (**f**) or NP-Ficoll (**g**), as assessed by NP-specific ELISA. (**h**) IgM and IgG1 NP-specific antibody responses of WT littermates and *Mb1^{cre}Hif2a$^{f/f}$* mice after immunization with NP-CGG, boosted with NP-CGG 5 weeks after primary immunization, as assessed by NP-specific ELISA. Arrowheads indicate the day of secondary immunization. Data are presented as mean±SD for 5–8 mice for each genotype at each time point. Pictures are representative of three (**a**,**b**) or two (**c**-**h**) independent experiments.

Supplementary Figure 3

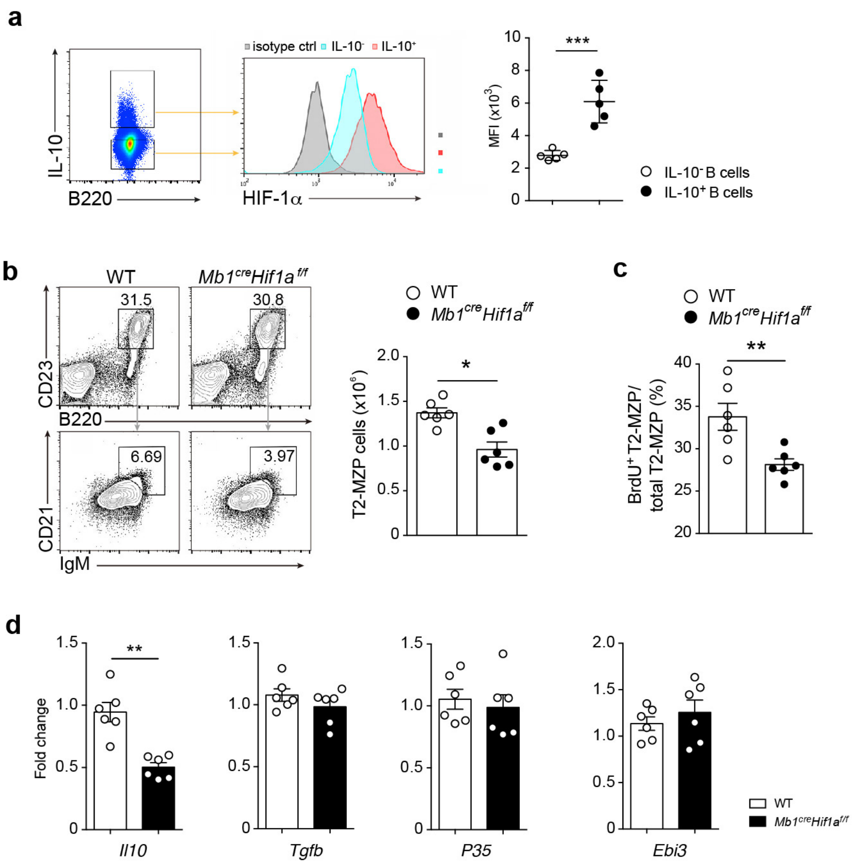

Supplementary Figure 3. Impaired T2-MZP B cells and reduced *Il10* mRNA expression in CD1d^{hi}CD5^{+} B cells from *Mb1^{cre}Hif1a$^{f/f}$* mice. (**a**) Flow cytometry-based quantification of HIF-1α expression in activated splenic B cells after gating on IL-10^{+} and IL-10^{-} B cells (n=5 per group). (**b**) Representative plots and absolute numbers of T2-MZP(CD19^{+}CD23^{hi}CD21^{hi}IgM^{+}) B cells in *Mb1^{cre}Hif1a$^{f/f}$* (n=6) and WT mice (n=6). (**c**) Percentage of BrdU^{+} cells in T2-MZP B cells from WT (n=6) and *Mb1^{cre}Hif1a$^{f/f}$* (n=6) mice 7 days after BrdU treatment. (**d**) Quantitative RT-PCR analyses of *Il10*, *Tgfb*, *P35* and *Ebi3* expression in sorted CD1d^{hi}CD5^{+} B cells from *Mb1^{cre}Hif1a$^{f/f}$* (n=6) and WT littermate mice (n=6). Data represent mean±s.e.m. Pictures are representative of three independent experiments. *P < 0.05, **P < 0.01 and ***P < 0.001 (unpaired, two-tailed Student's *t*-test).

Supplementary Figure 4

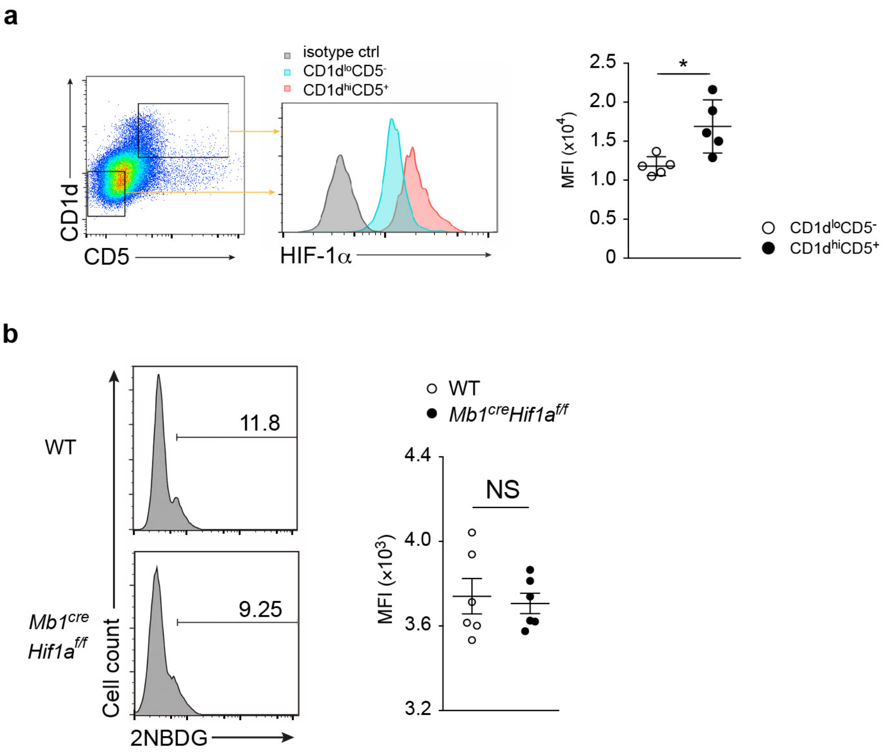

Supplementary Figure 4. High expression of HIF-1α in CD1d^{hi}CD5^{+} B cells. (**a**) Flow cytometry-based quantification of HIF-1α expression in splenic B cells after gating on CD1d^{hi}CD5^{+} and CD1d^{lo}CD5^{-} B cells (n=5 per group). (**b**) Glucose transport activity of sorted CD1d^{lo}CD5^{-} B cells from WT mice (n=6) and *Mb1^{cre}Hif1a$^{f/f}$* (n=6) measured by flow cytometry following with fluorescent glucose analog 2-NBDG. Data represent mean±s.e.m. Pictures are representative of three independent experiments. NS, not significant, *P < 0.05 (unpaired, two-tailed Student's *t*-test).

Supplementary Figure 5

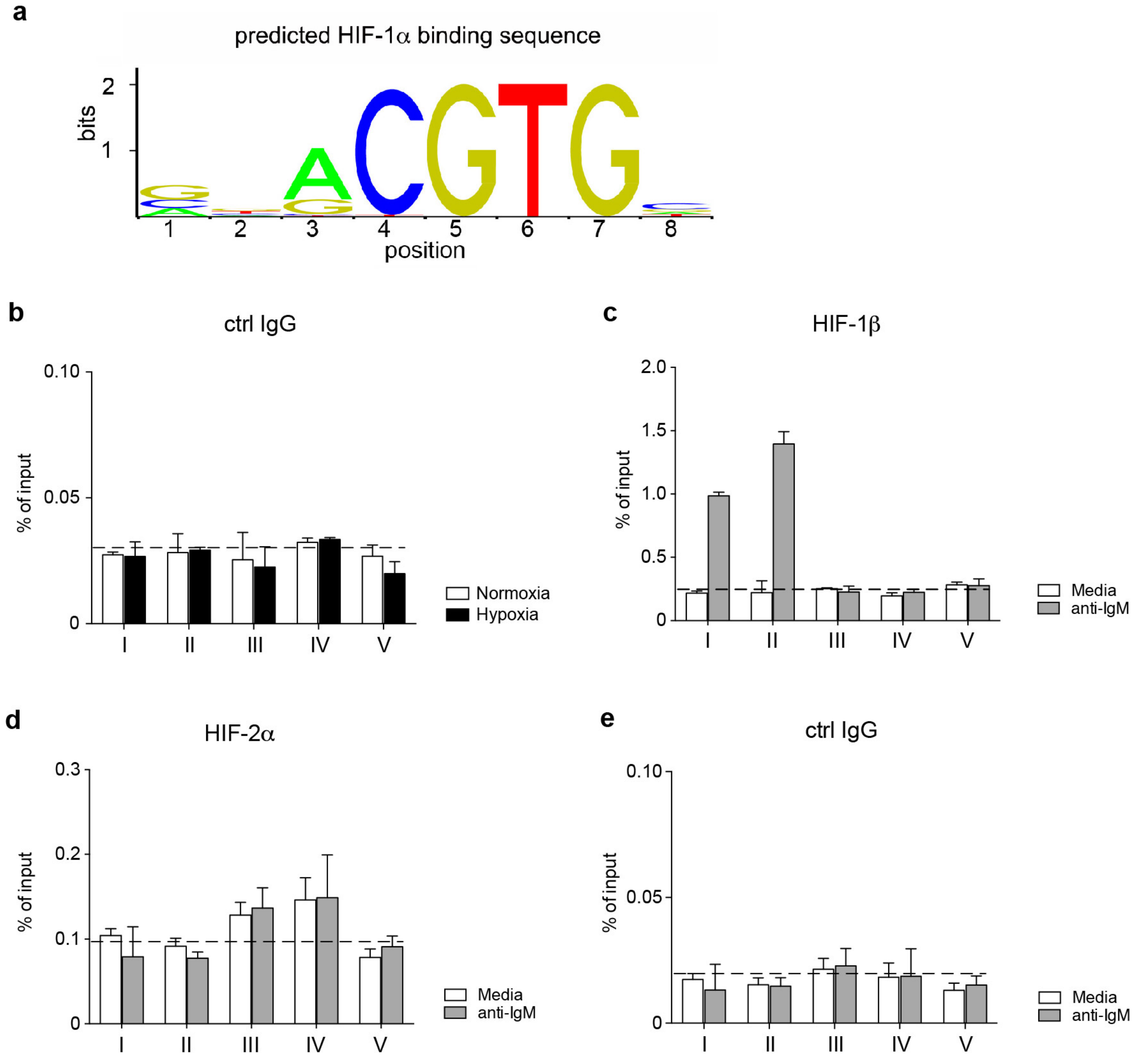

Supplementary Figure 5. HIF-1β, but not HIF-2α, binds to the *Il10* promoter. (**a**) Predicted HIF-1α binding sequence by JASPAR. (**b**) ChIP assays in enriched splenic B cells using control IgG (ctrl IgG) antibody on the HRE regions of *Il10* promoter under normoxia or hypoxia for 24 h (n=3 per group). (**c**-**e**) ChIP assays in enriched splenic B cells using HIF-1β (**c**), HIF-2α (**d**) and control IgG (ctrl IgG) (**e**) antibodies on the HRE regions of *Il10* promoter with or without anti-IgM stimulation for 8 h (n=3 for each group). Data represent mean±s.e.m. Pictures are representative of three independent experiments.

Supplementary Figure 6

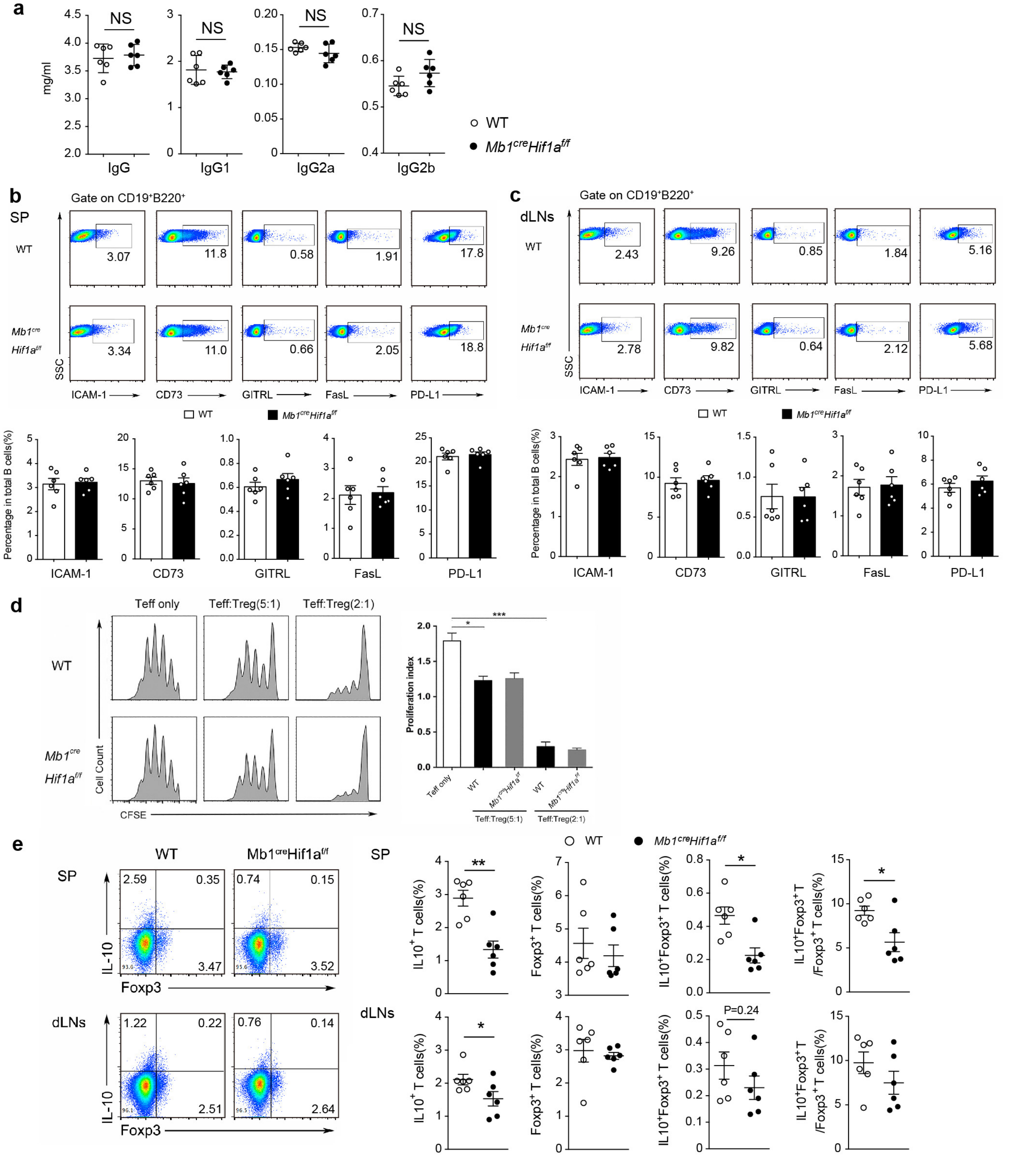

Supplementary Figure 6. Analysis of IL-10 independent mechanisms by B cells and IL-10⁺Foxp3⁺ T cells in *Mb1creHif1af/f* and WT mice after collagen immunization. (**a**) Levels of IgG, IgG1, IgG2a and IgG2b in the serum from *Mb1creHif1af/f* (n = 6) and WT mice (n = 6) after collagen immunization. (**b**,**c**) Representative plots and percentage of ICAM-1⁺,CD73⁺, GITRL⁺, FasL⁺ and PD-L1⁺ B cells in spleen (**b**) and dLNs (**c**) from *Mb1creHif1af/f* (n = 6) and WT mice (n = 6) after collagen immunization. (**d**) *In vitro* suppression assay of CD4⁺CD25⁺ T (Treg) cells isolated from spleen of WT littermates, *Mb1creHif1af/f*, and co-cultured for 72 h with CFSE-labeled CD4⁺CD25⁻T (Teff) cells in ratio of 1:2 or 1:5. Representative histogram plots (left) and statistical result of proliferation index on Teff cells (right, n=3 per group). (**e**) Representative plots and percentage of IL-10⁺CD4⁺, Foxp3⁺CD4⁺ and IL-10⁺Foxp3⁺CD4⁺ T cells in spleen and dLNs from *Mb1creHif1af/f* (n = 6) and WT mice (n = 6) after collagen immunization. Data represent mean±s.e.m. Pictures are representative of three independent experiments. NS, not significant, $*P < 0.05$, $**P < 0.01$ and $***P < 0.001$(unpaired, two-tailed Student's *t*-test).

Supplementary Figure 7

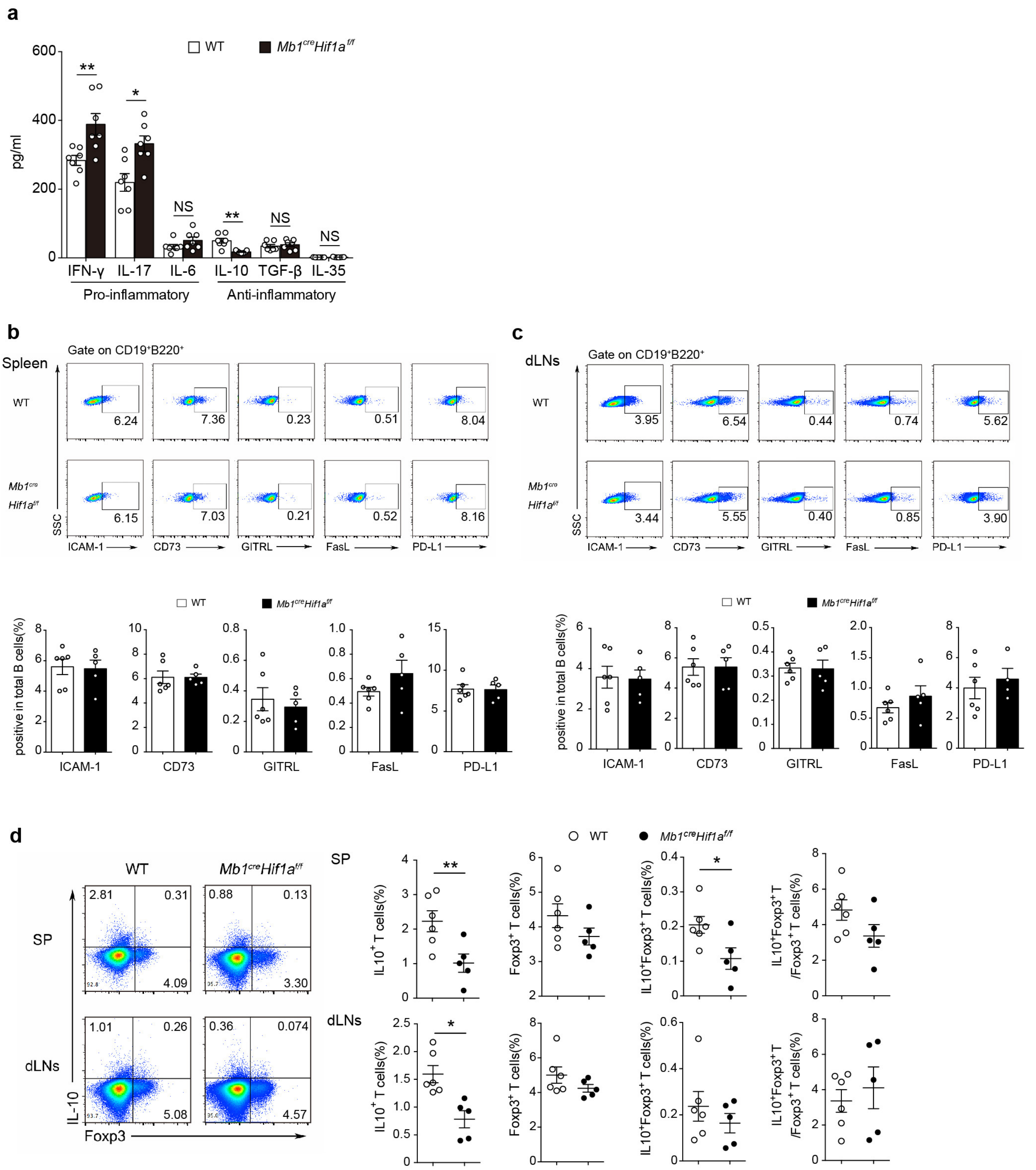

Supplementary Figure 7. Analysis of IL-10 independent mechanisms by B cells and IL-10⁺Foxp3⁺ T cells in *Mb1creHif1af/f* and WT mice after MOG immunization. (**a**) IFN-γ, IL-17, IL-6, IL-10, TGF-β and IL-35 levels in the serum of *Mb1creHif1af/f* (n = 7) and WT mice (n = 7) after MOG immunization. (**b**,**c**) Representative plots and percentage of ICAM-1⁺, CD73⁺, GITRL⁺, FasL⁺ and PD-L1⁺ B cells in spleen (**b**) and dLNs (**c**) from *Mb1creHif1af/f* (n = 5) and WT mice (n = 6) after MOG immunization. (**d**) Representative plots and percentage of IL-10⁺CD4⁺, Foxp3⁺CD4⁺ and IL-10⁺Foxp3⁺CD4⁺ T cells in spleen and dLNs from *Mb1creHif1af/f* (n = 5) and WT mice (n = 6) after MOG immunization. Data represent mean±s.e.m. Pictures are representative of three independent experiments. NS, not significant, *$P < 0.05$ and **$P < 0.01$ (unpaired, two-tailed Student's *t*-test).

Supplementary Figure 8

a

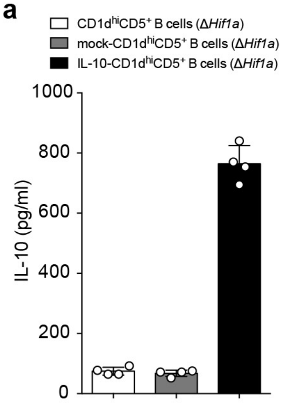

b

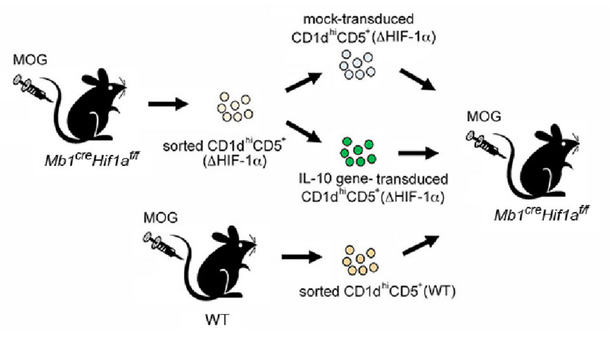

Supplementary Figure 8. IL-10 ecotopic-expression in *Hif1a*-deficient CD1d^{hi}CD5^{+} B cells and adoptive cell transfer in EAE model. (**a**) Sorted CD1d^{hi}CD5^{+} B cells from *Mb1^{cre}Hif1a$^{f/f}$* mice were transduced with pDBR (mock-CD1d^{hi}CD5^{+} B cells (*ΔHif1a*)) or pDBR-IL-10 (IL-10-CD1d^{hi}CD5^{+} B cells (*ΔHif1a*)), and IL-10 was quantified in culture supernatants at 24 h post-transduction (n=4 per group). CD1d^{hi}CD5^{+} B cells (*ΔHif1a*) are non-transduced CD1d^{hi}CD5^{+} B cells (*ΔHif1a*). Data represent mean±s.e.m. Pictures are representative of three independent experiments. (**b**) Schematic diagram of adoptive transfer of transduced CD1d^{hi}CD5^{+} B cells in EAE model.

Supplementary Figure 9

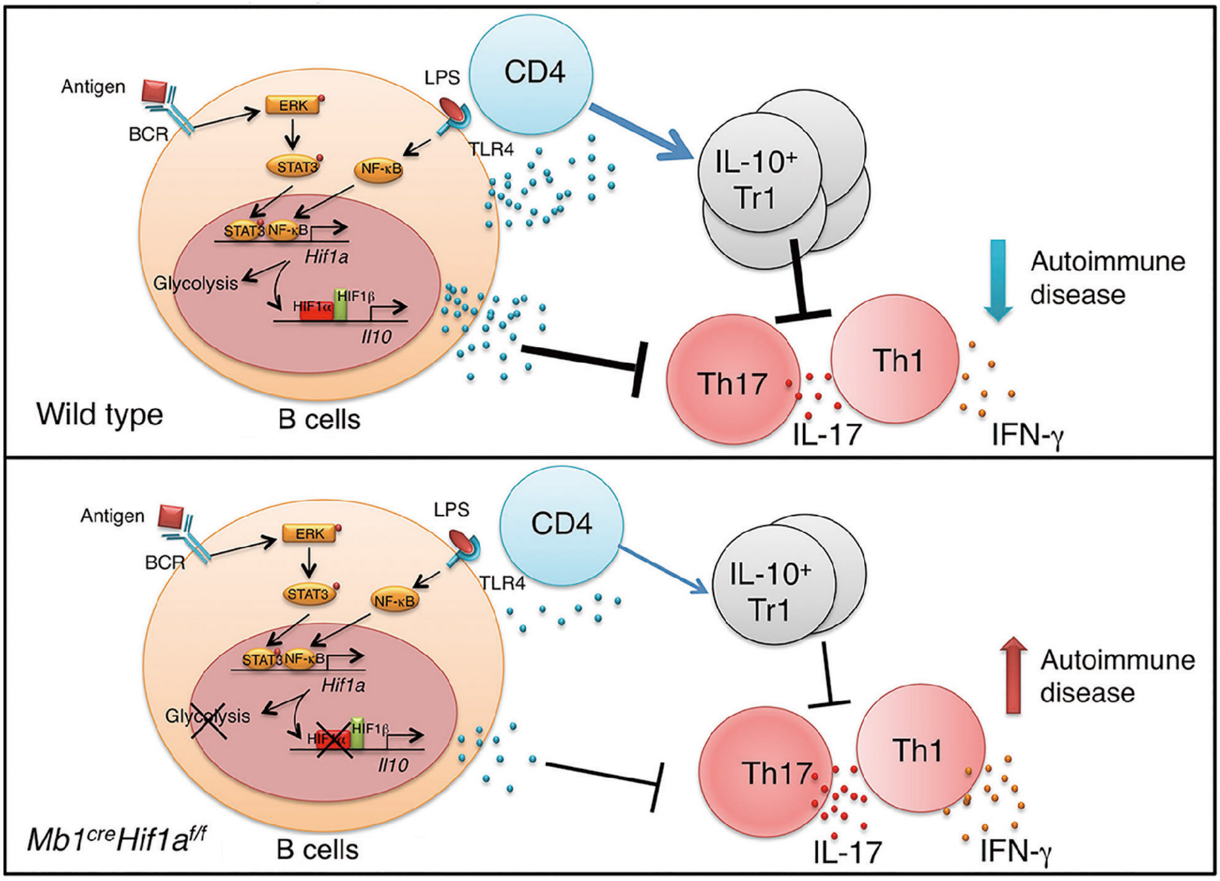

Supplementary Figure 9. Scheme model describing HIF-1α-dependent IL-10 production by B cells and how HIF-1α expression in B cells regulates autoimmune diseases such as experimental autoimmune encephalomyelitis and arthritis.

Supplementary Figure 10

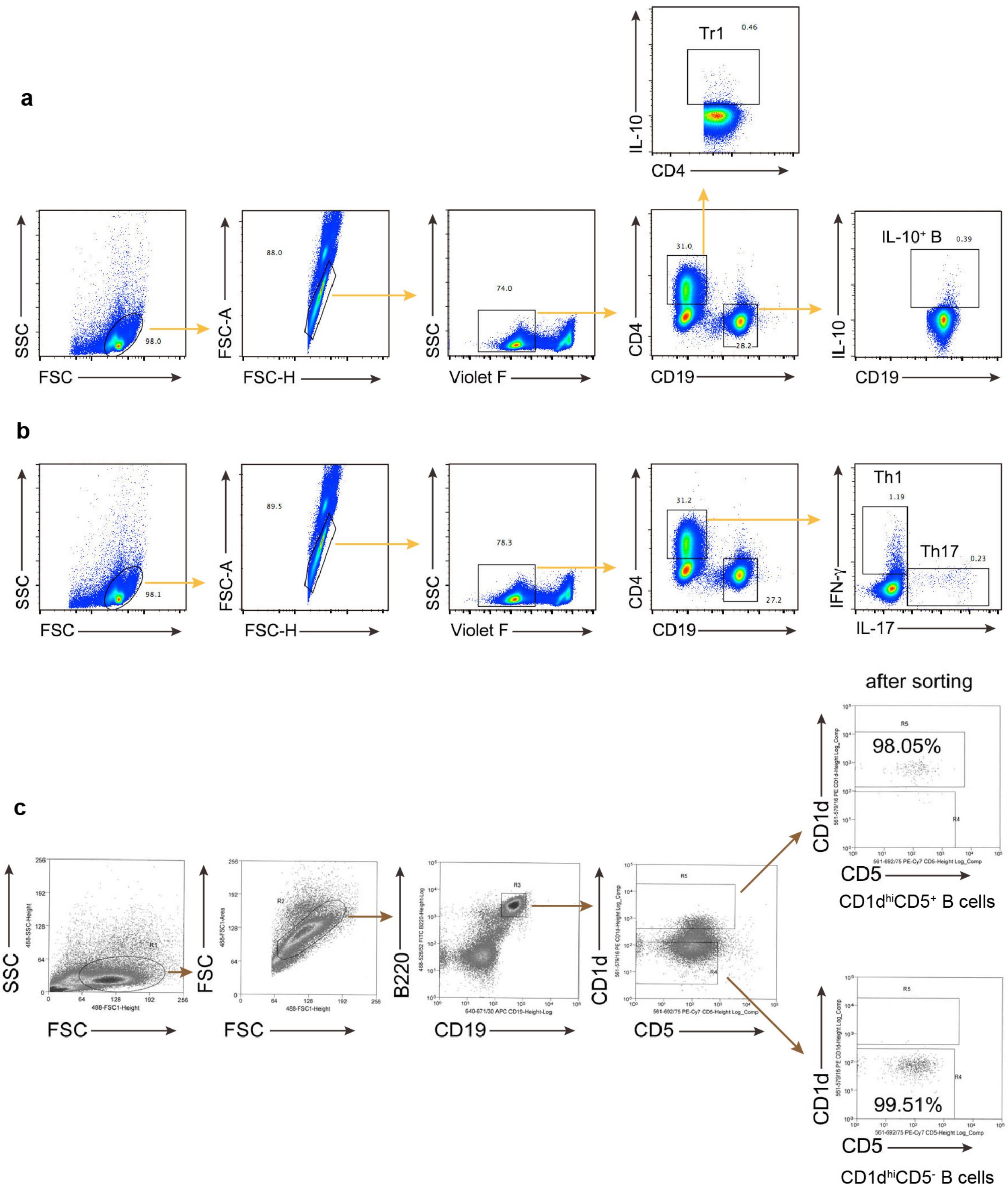

Supplementary Figure 10. Gating strategies for cytometry analysis and cell sorting. (**a**) Gating strategy for IL-10$^+$ B cells and Tr1 cells. (**b**) Gating strategy for Th1 and Th17 cells. (**c**) Gating strategy for cell sorting of CD1d^{hi}CD5$^+$ B cells and CD1d^{lo}CD5$^-$ B cells .

Supplementary Figure 11

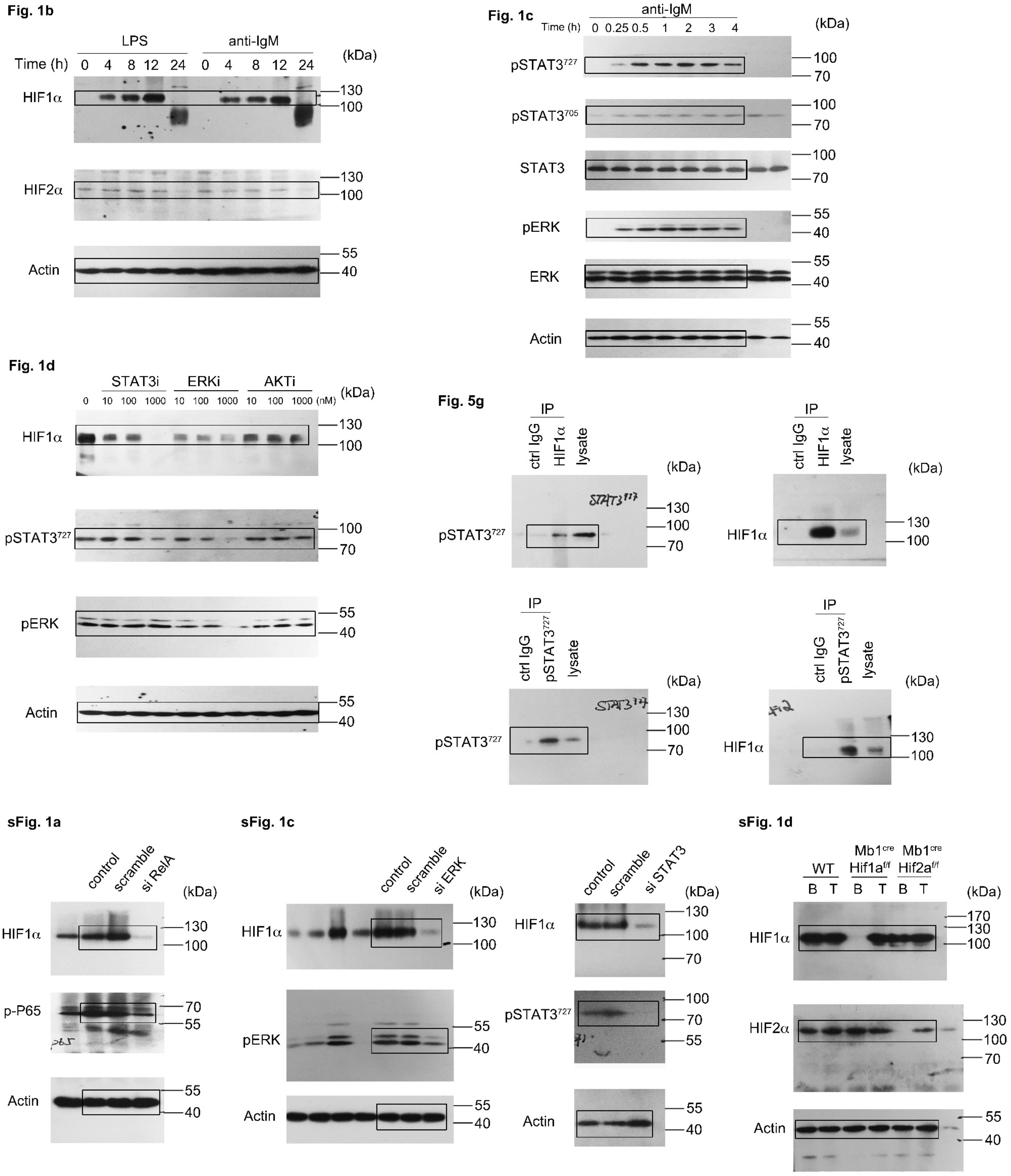

Supplementary Figure 11. Full scan of blots for figure 1b-d, figure 5g and supplementary figure 1a,c,d.

Supplementary table 1. List of antibodies and reagents

Antibodies

Protein detected	Source	Ig type (clone)	Application	Company
HIF-1α	Rabbit	Polyclonal(1006421)	WB;ChIP	Novus
HIF-1α	Mouse	IgG1 (H1a67)	WB;ChIP;Co-IP	Novus
β-actin	Mouse	IgG1 (A1978)	WB	Sigma
HIF-2α	Rabbit	Polyclonal (VB100-122)	WB	Novus
STAT3	Rabbit	Polyclonal (79D7)	WB	Cell Signaling Tech
Phospho-STAT3^{727}	Goat	Polyclonal (9134)	WB;Co-IP	Cell Signaling Tech
Phospho-STAT3^{705}	Goat	Polyclonal (9131)	WB	Cell Signaling Tech
ERK	Goat	Polyclonal (9102)	WB	Cell Signaling Tech
Phospho-ERK	Goat	Polyclonal (9101)	WB	Cell Signaling Tech
mouse IgG	Goat	Polyclonal (1036-05)	WB;Co-IP	Southern Biotech
rabbit IgG	Goat	Polyclonal (4055-05)	WB;Co-IP	Southern Biotech
CD3e	Rat	IgG2b (17A2)	Cell activation	ebiosciences
CD28	Rat	IgG2 (37.51)	Cell activation	ebiosciences
IL-4	Rat	IgG1 (11B11)	Cell activation	ebiosciences
IgM-F(ab)2	Goat	Mouse specific	Cell activation	Jackson ImmunoResearch
CD40	Rat	IgG2a (1C10)	Cell activation	ebiosciences
IgM	Rat	IgG2a (II/41)	Elisa capture	Southern Biotech
IgG1	Rat	IgG1 (A85-3)	Elisa capture	Southern Biotech
IgG3	Rat	IgG1 (R2-38)	Elisa capture	Southern Biotech
IgM	Rat	IgG2a (R6-60.2)	Elisa detection	Southern Biotech
IgG1	Rat	Rat IgG1 (A85-1)	Elisa detection	Southern Biotech
IgG3	Rat	IgG2a (R40-82)	Elisa detection	Southern Biotech
Isotype Control	Mouse	IgG1	ChIP	Thermo
Isotype Control	Rabbit	IgG, polyclonal	ChIP	Vector Laboratories
Histone H3 (tri methyl K4)	Rabbit	IgG, polyclonal	ChIP	Abcam
HIF-1β	Rabbit	IgG, polyclonal	ChIP	Cell Signaling Tech
IL-10	Rat	IgG2b (JES5-16E3)	Neutralization	Thermo
Isotype Control	Rat	IgG2b	Neutralization	Thermo

Protein detected	Conjugate	clone	Application and dilution	Company
BP-1	FITC	6C3	FACS(1/200)	ebioscience
CD21	PE	4E3	FACS(1/500)	ebioscience
CD11b	FITC	M1/70	FACS(1/200)	ebioscience
CD1d	FITC;APC	1B1	FACS(1/500;1/800)	ebioscience
CD4	APC	GK1.5	FACS(1/1,000)	ebioscience
F4/80	FITC	BM8	FACS(1/400)	ebioscience
IgD	FITC;PE-Cy5	11-26	FACS(1/200;1/500)	ebioscience
CD19	APC;PerCP-Cy5.5	1D3	FACS(1/500)	ebioscience
CD23	PE	B3B4	FACS(1/200)	ebioscience
IL-17A	PE	17B7	FACS(1/250)	ebioscience
CD24	PE	M1/69	FACS(1/200)	ebioscience
IL-10	PE;APC	JES-16E3	FACS(1/200)	ebioscience
CD25	FITC;PE-Cy5	PC61.5	FACS(1/500;1/800)	ebioscience
IFN-γ	PerCP-Cy5.5	XMG1.2	FACS(1/250)	ebioscience
CD93(AA4.1)	PE-Cy7	AA4.1	FACS(1/200)	biolegend
CD43	PE-Cy8	1B11	FACS(1/200)	biolegend
CD5	PE	53-7.3	FACS(1/450)	biolegend
B220	PerCP-Cy5.5	RA3-6B2	FACS(1/400)	biolegend
CD8	PE	53-6.7	FACS(1/1,500)	biolegend
IgM	APC	11E10	FACS(1/200)	southern biotech
Foxp3	APC	3G3	FACS(1/200)	mitenyi biotech
Isotype Control	PE	RTK4530	FACS(1/200)	biolegend
CD16/CD32		93	FACS(1/100)	biolegend
HIF-1α	PE	241812	FACS(1/100)	R&D
ICAM-1	FITC	YN1/1.7.4	FACS(1/200)	biolegend
CD73	PE	TY/11.8	FACS(1/200)	biolegend
GITRL	PE	YGL 386	FACS(1/200)	biolegend
FasL	PE	MFL3	FACS(1/200)	biolegend
PD-L1	PE	10F.9G2	FACS(1/200)	biolegend
IL-23R	PE	12B2B64	FACS(1/200)	biolegend
GM-CSF	PE	MP1-22E9	FACS(1/200)	biolegend
BrdU	FITC	BU20A	FACS(1/400)	ebioscience

Reagents				
**Name**		**Catalog Number**	**Company**	
Stattic		14590	cayman	
ERK inhibitor II		328007	Calbiochem	
AKT inhibitor VIII		14870	cayman	
PMA		P1585	Sigma	
Ionomycin		I0634	Sigma	
LPS		L4391	Sigma	
CFSE		C1157	invitrogen	
TOPRO		T3605	invitrogen	
polybrene		TR-1003-G	Millipore	
MOG peptide			Charité Berlin	
type II collagen		C9301	Sigma	
IL-2		200-02	peprotech	
IL-12		210-12	peprotech	
TGF-β		240-B-010	R&D	
IL-6		216-16	peprotech	
pertussis toxin		180	List/Quadra-tech	
NP-CGG		N-5055C	biosearch Technologies	
NP-Ficoll		F-1420	biosearch Technologies	
NP-BSA		N-5050H	biosearch Technologies	
2-DG		D8375	Sigma	
Celltrace violet		65-0840-85	ebioscience	
5-Bromo-2′-deoxyuridine		B5002	Sigma	

Supplemental table 2. List of primers

QPCR assay

Primer pair name	Sequence (5'-3')	Application used for	Comment
Hif1a (For)<br>*Hif1a* (Rev)	CCTGCACTGAATCAAGAGGTGC<br>CCATCAGAAGGACTTGCTGGCT	RT-qPCR	Intron spanning primers
Hif2a (For)<br>*Hif2a* (Rev)	GCGCTAGACTCCGAGAACAT<br>TGGCCACTTACTACCTGACCCTT	RT-qPCR	Intron spanning primers
Il10 (For)<br>*Il10* (Rev)	GCTGGACAACATACTGCTAACC<br>ATTTCCGATAAGGCTTGGCAA	RT-qPCR	Intron spanning primers
Tnf (For)<br>*Tnf* (Rev)	CACAGCCTTCCTCACAGAGC<br>GGAGGCAACAAGGTAGAGAGG	RT-qPCR	Intron spanning primers
Il17a (For)<br>*Il17a* (Rev)	CAGACTACCTCAACCGTTCCAC<br>TCCAGCTTTCCCTCCGCATTGA	RT-qPCR	Intron spanning primers
Ifng (For)<br>*Ifng* (Rev)	CAGCAACAGCAAGGCGAAAAAGG<br>TTTCCGCTTCCTGAGGCTGGAT	RT-qPCR	Intron spanning primers
Il1b (For)<br>*Il1b* (Rev)	TGGACCTTCCAGGATGAGGACA<br>GTTCATCTCGGAGCCTGTAGTG	RT-qPCR	Intron spanning primers
Hprt (For)<br>*Hprt* (Rev)	CTGGTGAAAAGGACCTCTCGAAG<br>CCAGTTTCACTAATGACACAAACG	RT-qPCR	Intron spanning primers
Glut1 (For)<br>*Glut1* (Rev)	AAGAAGCTGACGGGTCGCCTCATGC<br>TGAGAGGGACCAGAGCGTGGTG	RT-qPCR	Intron spanning primers
Pkm2 (For)<br>*Pkm2* (Rev)	CAGGAGTGCTCACCAAGTGG<br>CATCAAGGTACAGGCACTACAC	RT-qPCR	Intron spanning primers
Hk2 (For)<br>*Hk2* (Rev)	GGAGAGCACGTGTGACGAC<br>GATGCGACAGGCCACAGCA	RT-qPCR	Intron spanning primers
Ldha (For)<br>*Ldha* (Rev)	CACAAGCAGGTGGTGGACAG<br>AACTGCAGCTCCTTCTGGATTC	RT-qPCR	Intron spanning primers
Pdk1 (For)<br>*Pdk1* (Rev)	GATTCAGGTTCACGTCACGCT<br>GACGGATTCTGTCGACAGAG	RT-qPCR	Intron spanning primers
Gpi1 (For)<br>*Gpi1* (Rev)	GTTGCCTGAAGAGGCCAGG<br>GCTGTTGCTTGATGAAGCTGATC	RT-qPCR	Intron spanning primers

ChIP assay

Primer pair name	Sequence (5'-3')		
STAT3 (For)<br>STAT3 (Rev)	AGGTTACACGTCTCCAAGGC<br>CTCTGTGCTGTCGGGTAGAC		
HRE I (For)<br>HRE I (Rev)	TGATACGCCTGAGTGGCTGTCT<br>CACAAGAGCAGTGAGCGCTGAA		
HRE II (For)<br>HRE II (Rev)	GACATTGCCCTCCAGATCCC<br>TCTGTGCATTCCTTGGGGAC		
HRE III (For)<br>HRE III (Rev)	GGTCAACAGGACGTGTAGCA<br>ACATTCGCCTAGAGTCCCCT		
HRE IV (For)<br>HRE IV (Rev)	TTATGACCTGGGAGTGCGTG<br>AGCGCTAAAGAACTGGTCGG		
HRE V (For)<br>HRE V (Rev)	GAAAATCAGCCCTCTCGGGG<br>TTTCCTCCACTCAACCTGGG		

***Il10* Promoter regions cloning**

Primer pair name	Sequence (5'-3')		
HIF binding region I<br>(-3427_-3296)	CCGAGCTCTGGCAGCTGACTAGAAATAGGA<br>CCCTCGAGTTCCCTAGGATCAGGGAGGT		
HIF binding region II<br>(-2882_-2759)	CCGAGCTCTGGCAGCTGACTAGAAATAGGA<br>CCCTCGAGTTCCCTAGGATCAGGGAGGT		
HIF binding region III<br>(-1075_-951)	CCGAGCTCGGTCAACAGGACGTGTAGCA<br>CCCTCGAGACATTCGCCTAGAGTCCCCT		
HIF binding region IV<br>(-660_-540)	CCGAGCTCCTCTCCTCTGACCAACTGCC<br>CCCTCGAGCAAAGGAAACCCCGAGAGGG		
HIF binding region V<br>(-489_-369)	CCGAGCTCCAACCCAGGTTGAGTGGAGG<br>CCCTCGAGAGAGGCCCTCATCTGTGGAT		
